# Identifying Japanese Encephalitis Virus Using Metatranscriptomic Sequencing, Xinjiang, China

**DOI:** 10.3201/eid2806.210616

**Published:** 2022-06

**Authors:** Yi Yan, Rongjiong Zheng, Haizhou Liu, Zhiyong Wu, Mengchan Hao, Li Ma, Liying Wang, Jie Gao, Yining Yang, Di Liu, Xiaobo Lu

**Affiliations:** Wuhan Institute of Virology, Wuhan, China (Y. Yan, H. Liu, Z. Wu, M. Hao, L. Ma, L. Wang, J. Gao, D. Liu);; University of Chinese Academy of Sciences, Beijing, China (Y. Yan, Z. Wu, M. Hao, L. Ma, L. Wang, J. Gao, D. Liu);; First Affiliated Hospital of Xinjiang Medical University, Urumqi, China (R. Zheng, Y. Yang, D. Liu, X. Lu);; People's Hospital of Xinjiang Uygur Autonomous Region, Urumqi (Y. Yang).

**Keywords:** Japanese encephalitis virus, meningitis/encephalitis, metatranscriptomic sequencing, arboviruses, viruses, vector-borne infections, China

## Abstract

The treat of infectious disease epidemics has increased the critical need for continuous broad-ranging surveillance of pathogens with outbreak potential. Using metatranscriptomic sequencing of blood samples, we identified several cases of Japanese encephalitis virus infection from Xinjiang Uyghur Autonomous Region, China. This discovery highlights the risk for known viral diseases even in nonendemic areas.

Epidemics of infectious viral diseases seriously threaten human health and safety as well as social stability and development. In recent years, outbreaks of diseases such as influenza A(H7N9) (*1*), Ebola disease (*2*), and COVID-19 (*3*) have emphasized the need to continuously monitor potential pathogens in nature and pathogens known to circulate in human populations. Metagenomic sequencing (mNGS), because of its simplicity, low cost, and unbiased screening qualities, has been widely applied to identify pathogens for diagnosis and research. mNGS can simultaneously sequence multiple isolates, so it does not require anticipation of a specific disease cause, unlike conventional targeted-pathogen detection methods. mNGS can also analyze host immune response and the existence of antimicrobial resistance genes (*4*). 

We used mNGS for routine screening for tick-related pathogens in Xinjiang Uyghur Autonomous Region, China. We tested 10/25 retained blood samples collected from previously infected patients who had been bitten by ticks. Using Kraken2 (*5*), we preliminarily identified abundant amounts of 7 pathogens potentially associated with human disease: Proteus virus Isfahan, Japanese encephalitis virus (JEV), *Mycobacterium tuberculosis*, *Clostridium tetani*, hepatitis C virus (HCV), *Streptacidiphilus bronchialis*, and *Staphylococcus aureus* (Appendix Figure 1). At the same time, a de novo assembly used with BLAST-based methodology (https://blast.ncbi.nlm.nih.gov/Blast.cgi) found several human disease–related viruses: JEV, HCV, Crimean-Congo hemorrhagic fever virus, and human gammaherpesvirus 4. 

After we observed JEV and HCV in results from both methods, we confirmed presence of the 2 viruses based on results from a mapping-based method using Bowtie2 (*6*). The average coverage of JEV genome in 8 samples was 59.3%, and the coverage in sample 6 (GenBank accession no. MW766363) reached 99.5% (Appendix Figure 2). HCV was mapped only in sample 8 (GenBank accession no. MW766365). The results of JEV and HCV genome mapping were highly consistent with those using mNGS and the de novo assembly. 

Because of some inconsistent etiologic findings, we used different approaches to validate the presence of the other pathogens. Coverage of Crimean-Congo hemorrhagic fever virus reached an average of 15.6%, but the concentrated regions were highly homologous to human-sorting nexin 10. Coverage of human gammaherpesvirus 4 was negligible, <0.15%. Although Proteus virus Isfahan was abundant in reads, the signals detected were concentrated in regions 13608–13909 and 39231–39841, which had high similarity with human long noncoding RNA LHRI_lnC2063.9. As for the other 4 bacteria, we identified almost no homologous contigs by assembly, and the only contig related to *Staphylococcus aureus* in sample 10 was completely consistent with the origin sequence of *Homo sapiens* isolate CHM13 chromosome 21. The mapping approach showed <0.001 coverage of all 4 genomes and genome distribution was very scattered and low in depth (usually <10×) and showed no active expression of any specific genes. 

After gradual analysis and verification, we confirmed that there were JEV nucleic acids in some of these samples. Although confirming infection using JEV-specific antibody testing would have been ideal, extensive hemolysis of the samples precluded this testing. We reviewed the clinical manifestations of these patients, some of whom had fever, headache, and other signs and symptoms consistent only with mild JEV infections. 

Phylogenetic analysis of the JEV envelope gene showed that the viruses belonged to the G3 genotype (Figure), not G1, which has dominated epidemics since the 1950s (*7*). Because we did not rule out the history of vaccination among these patients and there is no JEV vaccination policy in Xinjiang, the genetic similarity between these strains and the G3 vaccine strain SA 14-14-2 suggested that vaccinated travelers might have imported (*7*) and shed the virus. Large numbers of imported JEV cases in nonendemic areas have been reported elsewhere (*8*). 

**Figure:**
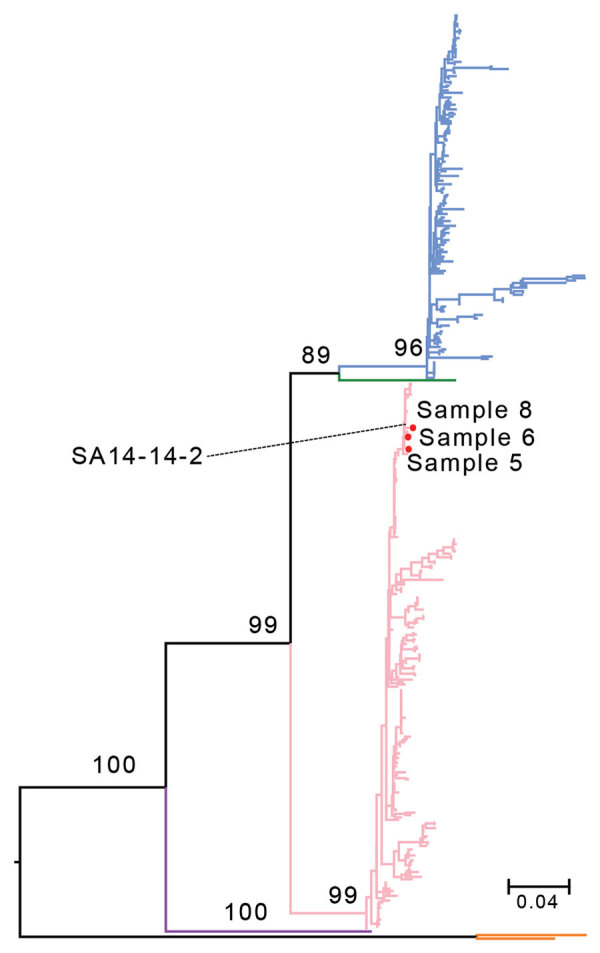
Maximum-likelihood tree based on the nucleic acid sequences of the Japanese encephalitis virus (JEV) envelope gene for 3 samples from persons in Xinjiang Province, China (samples 5, 6, and 8), and reference sequences. SA 14-14-2 is the JEV vaccine strain. The branch colors represent different JEV genotypes: blue branches indicate genotype 1; green, genotype 2; pink, genotype 3; purple, genotype 4; and orange, genotype 5. Values at nodes are bootstrap values supporting the branch. Scale bar indicates the substitution rate of equal-length branches.

These overlapping or inconsistent results might indicate some limitations in the use of a single method for metagenomic analysis, suggesting that although mNGS has been widely used, careful judgments are still necessary to avoid clinical misdiagnosis. In particular, when co-infection exists, such as JEV and HCV co-infection noted in this study, clinically misdiagnosing any pathogen might lead to serious medical consequences. Introducing unbiased mNGS testing into clinical practice should improve the rigor of analysis. 

Our study could not accurately determine the source or vectors of these JEV infections; ticks are not JEV hosts (*9*), and no mosquito species known to transmit JEV has been reported in Xinjiang (*10*). Despite our lack of information on the sources in these cases, our findings prompt us to strongly recommend strengthening surveillance for JEV and other emerging and reemerging pathogens in this region to prevent and neutralize the threat from pathogens before they cause public health incidents. 

Appendix Additional information about a study on identifying Japanese encephalitis virus using metatranscriptomic sequencing.
