# Supplementary material for: Identifying Japanese Encephalitis Virus Using Metatranscriptomic Sequencing, Xinjiang, China
Source: Emerg Infect Dis. 2022 Jun;28(6):1298–300. doi: 10.3201/eid2806.210616 (PMC9155892; doi:10.3201/eid2806.210616)
Supplement: Appendix — Additional information about a study on identifying Japanese encephalitis virus using metatranscriptomic sequencing. [file 21-0616-Techapp-s1.pdf]

Article DOI: https://doi.org/10.3201/eid2806.210616

Identifying Japanese Encephalitis Virus Using Metatranscriptomic Sequencing, Xinjiang Province, China

Appendix

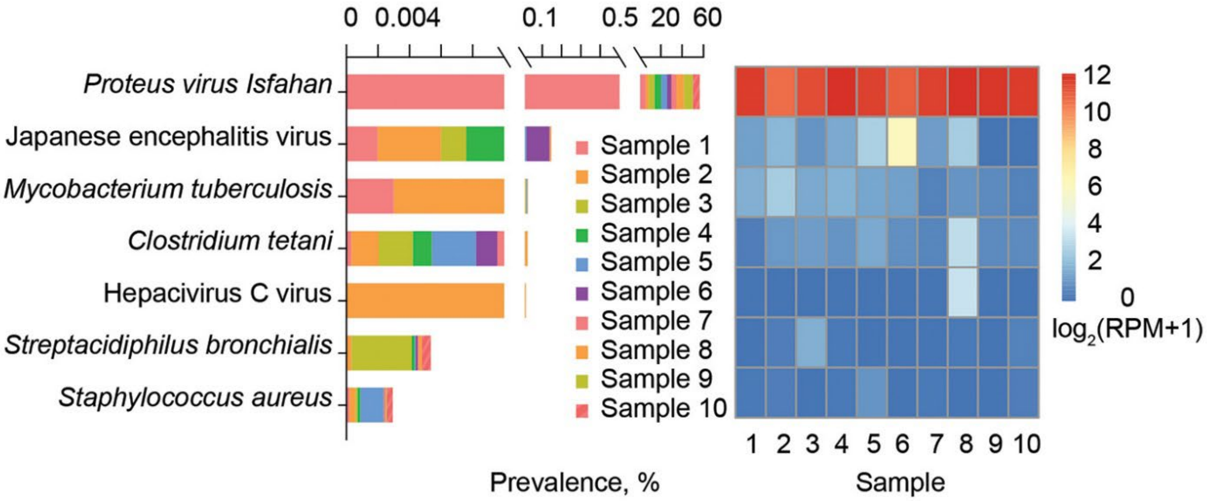

Appendix Figure 1. A) Prevalence and B) relative abundance of 7 potential pathogens in the study population. Prevalence indicates the proportion of each pathogenic reads/million nonhost reads. Side bar colors in panel B are keyed to abundance values. RPM: reads/million nonhost reads.

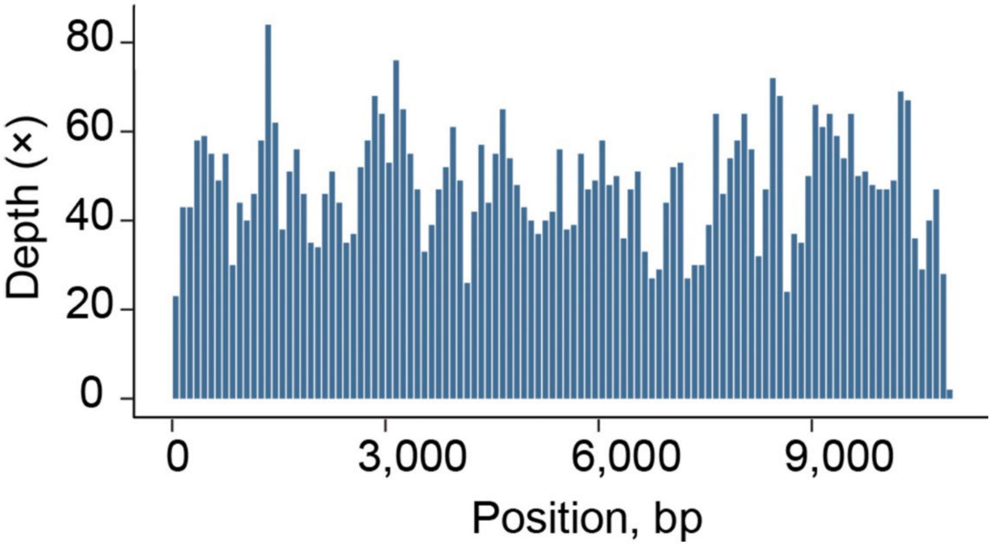

Appendix Figure 2. Genome coverage of Japanese encephalitis virus in sample 6 sequencing. The x axis represents genome position, and the y axis represents sequencing depth.
